# Purine Metabolism Alterations in Patients with Chronic Heart Failure: A Cross-Sectional Study of Associations with Iron Status, Oxidative Stress, and Anemia

**DOI:** 10.3390/metabo16060432

**Published:** 2026-06-22

**Authors:** Yessen Konysbek, Ayazhan Turar, Vilen B. Molotov-Luchanskiy, Olga A. Ponamareva

**Affiliations:** 1 Department of Internal Diseases, Karaganda Medical University, Karaganda 100028, Kazakhstan; molotov-luchanskiy@qmu.kz (V.B.M.-L.); ponamareva@qmu.kz (O.A.P.); 2 «OTAU MED» LLP, Shymkent 160021, Kazakhstan

**Keywords:** chronic heart failure, anemia, purine metabolism, adenine, oxidative stress, biomarkers, ejection fraction

## Abstract

**Background/Objectives**: Anemia and iron dysregulation are common in chronic heart failure (CHF), but additional metabolic mechanisms may contribute to these alterations. This study aimed to evaluate purine metabolism and oxidative stress markers in patients with CHF and to explore their potential relationship with anemia. **Methods**: In this cross-sectional study, 176 patients with CHF and 29 control individuals were included. CHF phenotypes were classified according to left ventricular ejection fraction (HFpEF, HFmrEF, HFrEF). Purine metabolites (guanine, hypoxanthine, adenine, xanthine, and uric acid) were measured using high-performance liquid chromatography, while lipid peroxidation (LPO) and advanced oxidation protein products (AOPPs) were assessed spectrophotometrically. Non-parametric statistical tests with correction for multiple comparisons were applied. **Results**: Anemia was present in 40.3% of patients with CHF. Serum iron and platelet counts were significantly lower in CHF compared with controls (*p* = 0.001). Among purine metabolites, adenine levels were higher in CHF (nominal *p* = 0.009), whereas other metabolites did not differ significantly between groups. LPO levels were lower and AOPP levels were higher in CHF (*p* = 0.021 and *p* = 0.008, respectively). No statistically significant associations were observed between hemoglobin levels and purine metabolites. **Conclusions**: CHF is associated with alterations in iron status and oxidative stress markers, as well as changes in purine metabolism. However, no significant associations between purine metabolites and anemia were identified in this cohort, and these findings should be interpreted cautiously given the exploratory design and sample size limitations.

## 1. Introduction

Chronic heart failure (CHF) remains one of the leading causes of morbidity and mortality worldwide, despite significant advances in pharmacological and device-based therapies [1]. Beyond impaired cardiac pump function, CHF is increasingly recognized as a systemic disorder characterized by metabolic dysregulation, chronic inflammation, and multiorgan involvement [2]. Among the most clinically relevant comorbidities of CHF, anemia occupies a central position due to its high prevalence, negative prognostic impact, and contribution to reduced exercise tolerance, frequent hospitalizations, and increased mortality [3]. Anemia is detected in up to 30–70% of patients with CHF, depending on disease severity, comorbid conditions, and diagnostic criteria [4]. Its pathogenesis is multifactorial and includes functional iron deficiency mediated by hepcidin overexpression, impaired erythropoietin synthesis in the setting of renal dysfunction, chronic low-grade inflammation, bone marrow suppression, and hemodilution [5]. Although iron deficiency and inflammatory pathways have been extensively studied, they do not fully explain the heterogeneity of anemia in CHF or the variable response to iron therapy. While intravenous iron improves functional capacity and reduces hospitalizations in some patients, effects on mortality are inconsistent and oral iron often provides limited benefit, suggesting the involvement of additional metabolic mechanisms [6,7]. This illustrates the need to identify additional metabolic contributors involved in the development and persistence of anemia in CHF.

In recent years, increasing attention has been directed toward disturbances of cellular energy metabolism in CHF. Chronic myocardial hypoxia, impaired mitochondrial function, and reduced oxidative phosphorylation lead to accelerated adenosine triphosphate (ATP) degradation [8,9]. As a consequence, purine metabolism is activated, resulting in the accumulation of purine catabolites such as hypoxanthine, xanthine, adenine, and uric acid [10]. These metabolites are not merely passive by-products of energy depletion but actively participate in redox imbalance through xanthine oxidase activation, generation of reactive oxygen species, and amplification of oxidative stress [11]. Oxidative stress plays a pivotal role in CHF progression and is closely linked to inflammation, endothelial dysfunction, and impaired iron homeostasis [12]. Experimental and clinical data suggest that oxidative stress and purine metabolites may influence erythropoiesis by suppressing erythropoietin signaling, altering iron mobilization, and promoting the inflammatory blockade of iron utilization [13,14]. However, despite growing evidence connecting purine metabolism to cardiovascular remodeling and oxidative injury, its specific contribution to anemia development in CHF remains insufficiently explored [15]. Importantly, CHF is a heterogeneous syndrome encompassing distinct phenotypes based on left ventricular ejection fraction (LVEF), including heart failure with preserved (HFpEF), mildly reduced (HFmrEF), and reduced ejection fraction (HFrEF). These phenotypes differ not only in hemodynamic characteristics but also in inflammatory burden, metabolic profiles, and systemic consequences [16,17]. Whether purine metabolism disturbances and their association with anemia vary across CHF phenotypes remains unclear.

In this context, we hypothesized that disturbances in purine metabolism represent a systemic metabolic feature of CHF that contributes to oxidative stress and interacts with iron metabolism and hematopoiesis, thereby influencing the development and severity of anemia. We further postulated that specific purine metabolites may serve as potential biochemical markers reflecting metabolic overload and inflammatory anemia in CHF. The aim of this study was to evaluate the relationship between purine metabolism disturbances, oxidative stress markers, and anemia in patients with chronic heart failure, with particular emphasis on differences across LVEF-based CHF phenotypes. By integrating purine profiling with hematological and oxidative stress parameters, we sought to clarify the metabolic background of anemia in CHF and to identify candidate biomarkers with potential diagnostic and pathophysiological relevance.

## 2. Methods

### 2.1. Study Design and Ethics

This observational, cross-sectional study was conducted between June 2022 and August 2023 at City Polyclinic No. 1 and the clinic of NJSC Karaganda Medical University (KMU), Karaganda, Kazakhstan. The study protocol was approved by the Local Ethics Committee of NJSC Karaganda Medical University (Protocol No. 11, 22 April 2022; registration No. 58) and was carried out in accordance with the Declaration of Helsinki and national regulatory requirements. All participants provided written informed consent prior to enrollment.

### 2.2. Study Population

Adult patients (≥18 years) with a confirmed diagnosis of CHF were consecutively enrolled during routine clinical visits. CHF was diagnosed in accordance with the Clinical Protocol of the Ministry of Health of the Republic of Kazakhstan, based on clinical assessment and transthoracic echocardiography. Patients were classified according to left ventricular ejection fraction (LVEF) into heart failure with preserved ejection fraction (HFpEF; LVEF ≥ 50%), heart failure with mildly reduced ejection fraction (HFmrEF; LVEF 41–49%), and heart failure with reduced ejection fraction (HFrEF; LVEF ≤ 40%). Anemia was defined according to World Health Organization criteria as hemoglobin <130 g/L in men and <120 g/L in women. The study size was determined by the number of eligible patients with chronic heart failure attending the participating centers during the study period, along with available control participants meeting the inclusion criteria. Because chronic heart failure predominantly affects older adults, no age restrictions beyond adulthood (≥18 years) were applied. Age was subsequently considered as a potential confounding variable and included in multivariable analyses.

Patients were excluded if they had acute decompensated heart failure or cardiogenic shock, active infectious or inflammatory disease, end-stage chronic kidney disease requiring dialysis, active malignancy, myocardial infarction or stroke within the previous 3 months, or hematological disorders unrelated to CHF. Patients with active infectious or inflammatory diseases, active malignancy, hematological disorders unrelated to CHF, and end-stage chronic kidney disease requiring dialysis were excluded in order to reduce the influence of conditions known to substantially affect oxidative stress, iron metabolism, and purine metabolism. The control group consisted of 29 individuals without clinical symptoms or echocardiographic evidence of CHF. Control participants were recruited from individuals attending routine health examinations at the same institutions during the same period and were selected to have no clinical or echocardiographic evidence of CHF. The control group consisted of community-dwelling adults recruited from individuals undergoing routine preventive health examinations at the participating institutions during the study period. To improve comparability with the CHF cohort, control participants were selected from the same geographic region and healthcare settings. Individuals were eligible for inclusion only if they had no clinical symptoms or echocardiographic evidence of CHF and no history of major cardiovascular disease. Participants with active infectious or inflammatory diseases, active malignancy, end-stage renal disease, hematological disorders, autoimmune diseases, or other conditions known to substantially affect purine metabolism, iron homeostasis, oxidative stress markers, or hematological parameters were not eligible for inclusion. Age and sex were recorded for all participants and were considered as potential confounding variables in the statistical analysis. Randomization was not performed due to the observational nature of the study.

To minimize selection bias, patients were consecutively enrolled during routine clinical visits, and control participants were recruited from the same institutions during the same period. Measurement bias was reduced by using standardized laboratory protocols and performing all analyses in a single certified laboratory. Potential confounding was partially addressed through multivariable regression analysis adjusting for age, sex, LVEF category, and anemia status. However, information regarding several clinically relevant comorbidities and medication use was not systematically available for all participants and therefore could not be incorporated into the regression model.

The purpose of the regression analysis was to determine whether the observed differences in adenine concentrations were independently associated with demographic and clinical variables after adjustment for potential confounders. In this model, the regression coefficient (β) represents the estimated change in adenine concentration associated with a one-unit increase in a continuous variable or with membership in a given categorical group, while holding the remaining variables constant. The 95% confidence interval (CI) was used to assess the precision and statistical significance of each estimate.

The control group size was limited by the availability of eligible individuals without CHF during the study period. Therefore, the analysis should be considered exploratory, particularly for between-group comparisons.

### 2.3. Clinical and Laboratory Assessments

Transthoracic echocardiography was performed by a certified cardiologist using a General Electric Logiq P9 ultrasound system (GE Healthcare, Chicago, IL, USA). LVEF was calculated using the biplane Simpson’s method in accordance with European Society of Cardiology recommendations. Venous blood samples were collected in the morning after an overnight fast. Hemoglobin concentration, platelet count, serum iron, and ferritin levels were measured using an automated hematology analyzer (Sysmex KX-21N, Sysmex Corporation, Kobe, Japan) and a biochemical analyzer (Beckman Coulter AU480, Beckman Coulter Inc., Brea, CA, USA). Plasma and erythrocyte concentrations of purine metabolites (adenine, hypoxanthine, xanthine, guanine, and uric acid) were quantified by high-performance liquid chromatography (HPLC) with ultraviolet detection using a modified and validated analytical protocol based on previously published methods. Markers of oxidative stress, including lipid peroxidation (LPO) products and advanced oxidation protein products (AOPPs), were assessed spectrophotometrically using a PD-303UV spectrophotometer (APEL Co., Ltd., Saitama, Japan). All analyses were performed in the certified clinical–biochemical laboratory of NJSC Karaganda Medical University.

The primary outcomes of interest were purine metabolite concentrations and oxidative stress markers. Chronic heart failure status and anemia were considered exposure variables, while age, sex, and LVEF category were treated as potential confounders. All laboratory measurements were performed using the same standardized protocols and equipment for both patients and control participants to ensure comparability between groups.

### 2.4. Statistical Analysis

Statistical analyses were performed using Minitab version 19 (Minitab LLC, State College, PA, USA). Data distribution was assessed using the Anderson–Darling test. Continuous variables are presented as median and interquartile range (IQR). Comparisons between two groups were performed using the Mann–Whitney U test, while comparisons among three or more groups were conducted using the Kruskal–Wallis test. When statistically significant differences were identified, post hoc pairwise comparisons were performed using Dunn’s test with Holm adjustment. To control for multiple comparisons, *p*-values were adjusted using the Holm–Bonferroni method. All statistical tests were two-sided. Statistical significance was defined as *p* ≤ 0.05. Missing data were handled using complete-case analysis, as the proportion of missing data was minimal. No formal sample size or power calculation was performed due to the exploratory nature of the study and real-world recruitment constraints. LVEF categories were defined according to established European Society of Cardiology guidelines.

To further assess independent associations with adenine levels, a multivariable linear regression analysis was performed. Adenine concentration was included as the dependent variable, while age, sex, LVEF category, and anemia status were entered as independent variables. Age was specifically included in the model to account for its potential confounding effect on purine metabolism, iron homeostasis, oxidative stress markers, and hematological parameters. Regression coefficients (β) with 95% confidence intervals (CI) were calculated. Model fit was assessed using the coefficient of determination (R^2^). Statistical significance was defined as *p* ≤ 0.05. Given the sample size and exploratory design, regression results were interpreted with caution. No sensitivity analyses or age-stratified analyses were performed; therefore, residual confounding related to age and age-associated comorbidities cannot be completely excluded.

Assumptions of the linear regression model were evaluated by inspection of residual plots and assessment of residual distribution. Because several study variables demonstrated non-normal distributions, the regression analysis was considered exploratory and interpreted cautiously.

## 3. Results

### 3.1. Study Population Characteristics

A total of 176 patients with CHF were included in the analysis, comprising 105 men (59.7%) and 71 women (40.3%). Anemia was present in 71 patients (40.3%). Patients were classified according to left ventricular ejection fraction into HFpEF (*n* = 51), HFmrEF (*n* = 64), and HFrEF (*n* = 61) groups. The mean age of patients with CHF was 69.4 ± 10.5 years. A control group of 29 individuals without CHF was included for comparative analyses. Missing data were minimal and did not materially affect the analysis.

### 3.2. Comorbidities Across CHF Phenotypes

The prevalence of selected cardiovascular comorbidities differed significantly across CHF phenotypes. Ischemic heart disease was most frequent in patients with HFmrEF (98.5%) compared with HFpEF (67.9%) and HFrEF (61.3%) (*p* < 0.001). Atrial fibrillation occurred more commonly in HFmrEF (44.6%) and HFrEF (38.7%) than in HFpEF (11.3%) (*p* < 0.01). Mitral and tricuspid valve defects were significantly more prevalent in HFmrEF and HFrEF than in HFpEF (*p* = 0.015 and *p* = 0.009, respectively). Three-vessel coronary artery disease was the most frequent in HFmrEF (36.9%) (*p* = 0.048). The prevalence of chronic kidney disease was the highest in HFrEF (30.7%), although this difference did not reach statistical significance.

### 3.3. Comparison Between CHF Patients and Controls

Several differences were observed between patients with CHF and control group in several biochemical and hematological parameters (Table 1). Serum iron levels were significantly lower in CHF patients compared with controls (11.5 [6.9–17.5] vs. 16.2 [13.5–19.8] µmol/L, *p* = 0.001), while platelet counts were also reduced (225 [186.0–269.5] vs. 288 [234.8–315.0] × 10^9^/L, *p* = 0.001).

Among purine metabolism markers, adenine levels were higher in CHF patients (nominal *p* = 0.009), whereas guanine, hypoxanthine, and xanthine did not differ significantly between groups (all *p* > 0.05). Markers of oxidative stress also differed significantly. LPO were lower in CHF patients (0.157 [0.119–0.258] vs. 0.215 [0.175–0.242], *p* = 0.021), while AOPPs were higher (0.194 [0.135–0.277] vs. 0.133 [0.086–0.200], *p* = 0.008). No significant differences were observed for ferritin, hemoglobin, or uric acid levels between the groups.

In the multivariable regression analysis, sex was identified as the only independent factor significantly associated with adenine concentrations (β = 30.02, 95% CI: 9.33–50.70, *p* = 0.0047). This finding suggests that sex-related biological differences may contribute to variability in purine metabolism among patients with CHF. Although the present study was not specifically designed to evaluate sex-dependent metabolic profiles and formal sex-stratified analyses were not performed, the observed association indicates that sex may represent an important determinant of adenine metabolism and warrants further investigation in future studies.

Spearman correlation analysis performed in the CHF population (*n* = 176) did not reveal significant associations between hemoglobin levels and purine metabolites, including guanine (r = 0.066, *p* = 0.383), hypoxanthine (r = 0.076, *p* = 0.316), adenine (r = 0.059, *p* = 0.438), and xanthine (r = 0.088, *p* = 0.248).

### 3.4. Laboratory Parameters Across CHF Phenotypes

Significant differences in several laboratory parameters were observed across CHF phenotypes defined by left ventricular ejection fraction (Table 2). Serum iron levels differed significantly between groups, with lower values in HFrEF compared to HFmrEF (*p* < 0.05), while HFpEF showed intermediate values without significant differences from the other groups. Ferritin levels were significantly higher in HFrEF compared to HFpEF and HFmrEF (*p* < 0.05), suggesting more pronounced alterations in iron metabolism in patients with reduced ejection fraction. Hemoglobin levels did not differ significantly between groups. However, platelet counts were significantly lower in HFrEF compared to HFpEF and HFmrEF (*p* < 0.05), indicating potential differences in hematological status associated with disease severity. Although median hemoglobin concentrations were comparable across CHF phenotypes, anemia was present in 40.3% of the overall CHF cohort according to WHO diagnostic criteria. Therefore, the absence of significant differences in continuous hemoglobin values between HF phenotypes should not be interpreted as evidence of a low prevalence of anemia within the study population. No statistically significant differences were observed in total purine levels, hypoxanthine, adenine, xanthine, or uric acid across CHF phenotypes. Similarly, markers of oxidative stress, including LPO and AOPP, did not show significant variation between groups.

### 3.5. Comparison of Laboratory Parameters in CHF Patients with and Without Anemia

Comparative analysis of laboratory parameters between CHF patients with anemia, CHF patients without anemia, and the control group revealed several statistically significant differences (Table 3). Serum iron levels were significantly lower in CHF patients with anemia compared to both CHF patients without anemia and controls (7.1 µmol/L vs. 13.8 µmol/L and 16.2 µmol/L, respectively; *p* < 0.05). Similarly, hemoglobin levels were markedly reduced in the anemia subgroup (106.0 g/L) compared to CHF patients without anemia (144.0 g/L) and controls (132.0 g/L), reflecting the expected hematological profile. Platelet counts were significantly higher in the control group compared to CHF patients without anemia, while intermediate values were observed in CHF patients with anemia. Ferritin levels were elevated in CHF patients without anemia compared to controls, although no statistically significant difference was observed between CHF patients with anemia and the other groups. Regarding purine metabolism, adenine levels were significantly higher in both CHF subgroups compared to controls, suggesting enhanced purine turnover in CHF irrespective of anemia status. In contrast, no significant differences were observed for total purines, hypoxanthine, xanthine, or uric acid between groups.

Markers of oxidative stress demonstrated distinct patterns. AOPP levels were significantly elevated in CHF patients without anemia compared to controls, while LPO levels did not differ significantly between CHF subgroups but were lower compared to controls.

### 3.6. Laboratory Parameters Across CHF Phenotypes Stratified by Anemia Status

**Figure 1:**
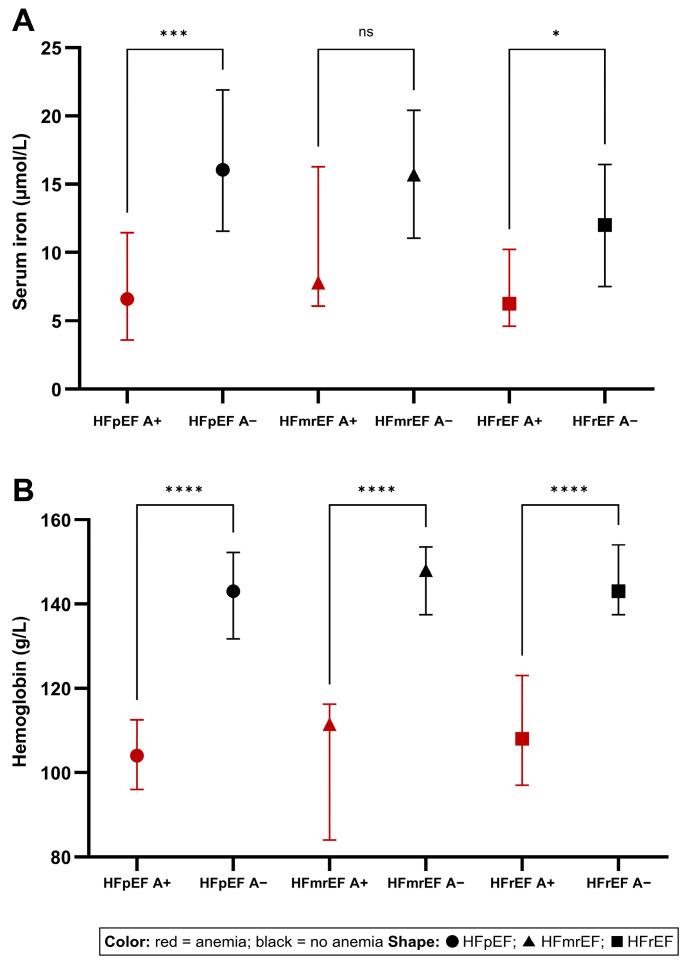
Serum iron and hemoglobin levels across CHF phenotypes stratified by anemia status. (**A**) Serum iron and (**B**) hemoglobin levels in CHF patients stratified by phenotype (HFpEF, HFmrEF, HFrEF) and anemia status (A+, red; A−, black). Data are presented as median (IQR). Symbols denote phenotypes (● HFpEF; ▲ HFmrEF; ■ HFrEF). Statistical analysis was performed using the Kruskal–Wallis test with Dunn’s post hoc test. Brackets indicate comparisons between anemia and non-anemia within each phenotype. * *p* < 0.05; *** *p* < 0.001; **** *p* < 0.0001; ns—not significant.

A detailed analysis of laboratory parameters stratified by both CHF phenotype and anemia status is presented in Supplementary Table S1. Across all phenotypes, patients with anemia consistently demonstrated lower serum iron and hemoglobin levels compared to those without anemia (Figure 1). This pattern was observed in HFpEF, HFmrEF, and HFrEF groups, did not demonstrate statistically significant differences across subgroups. Within the HFpEF group, serum iron levels were significantly lower in patients with anemia compared to those without anemia (*p* < 0.001), with a similar difference observed in HFrEF (*p* < 0.05), while no significant difference was found in HFmrEF. Hemoglobin levels were significantly reduced in patients with anemia across all CHF phenotypes (*p* < 0.0001 for all comparisons) (Figure 1B).

Ferritin levels tended to be higher in patients without anemia, although this pattern was not consistent across all phenotypes. Platelet counts showed moderate variation between subgroups without a clear trend. No statistically significant differences were observed between subgroups for total purines, hypoxanthine, adenine, xanthine, or uric acid, suggesting that purine metabolism is not substantially influenced by anemia status within CHF phenotypes. Similarly, oxidative stress markers (LPO and AOPP) did not demonstrate statistically significant differences across subgroups.

Overall, these findings indicate that anemia primarily affects iron-related parameters, while purine metabolism and oxidative stress markers did not show statistically significant differences according to anemia status across CHF phenotypes.

### 3.7. Correlation Analysis

Spearman correlation analysis was performed to evaluate associations between hematological parameters, iron metabolism markers, purine metabolites, and oxidative stress indicators within the CHF population. Given the number of correlations tested, results should be interpreted cautiously due to the potential for type I error.

In the overall CHF cohort (*n* = 176), a positive correlation was observed between hemoglobin and serum iron levels (r = 0.469, *p* < 0.001). A weaker correlation was also found between ferritin and serum iron (r = 0.195, *p* = 0.009). No significant correlations were detected between hemoglobin levels and purine metabolites, including guanine (r = 0.066, *p* = 0.383), hypoxanthine (r = 0.076, *p* = 0.315), adenine (r = 0.059, *p* = 0.438), and xanthine (r = 0.088, *p* = 0.248). In the subgroup of CHF patients with anemia (*n* = 71), hemoglobin levels demonstrated a positive correlation with serum iron (r = 0.307, *p* = 0.009). However, no significant associations were observed between hemoglobin and purine metabolites in this subgroup (all *p* > 0.05). Ferritin levels were positively correlated with several purine metabolites, including guanine (r = 0.316, *p* = 0.007), hypoxanthine (r = 0.347, *p* = 0.003), adenine (r = 0.362, *p* = 0.002), and xanthine (r = 0.389, *p* = 0.001).

In CHF patients without anemia (*n* = 105), hemoglobin levels were not significantly associated with serum iron or purine metabolites (all *p* > 0.05). However, positive correlations were observed between the oxidative stress marker AOPP and several purine metabolites, including guanine (r = 0.390, *p* < 0.001), hypoxanthine (r = 0.402, *p* < 0.001), adenine (r = 0.368, *p* < 0.001), and xanthine (r = 0.291, *p* = 0.003).

### 3.8. Multivariable Analysis of Factors Associated with Adenine Levels

To further evaluate factors associated with adenine levels, a multivariable linear regression analysis was performed including age, sex, LVEF category, and anemia status as independent variables (Table 4). In this model, only sex showed a statistically significant association with adenine levels (β = 30.02, 95% CI: 9.33 to 50.70, *p* = 0.0047), with higher levels observed in males. No significant associations were found for age (*p* = 0.4346), LVEF category (*p* = 0.6932), or anemia status (*p* = 0.7570).

The model demonstrated low explanatory power (R^2^ = 0.049), indicating that these clinical variables explain only a small proportion of the variability in adenine levels, and results should be interpreted cautiously given the exploratory design and deviation from normality of residuals. To determine whether adenine concentrations were independently associated with selected demographic and clinical characteristics, a multivariable linear regression analysis was performed. Adenine concentration was entered as the dependent variable, while age, sex, LVEF category, and anemia status were included as independent variables. The results are presented in Table 4.

The multivariable regression model explained only 4.9% of the variability in adenine concentrations (R^2^ = 0.049), indicating limited overall explanatory power. Among the variables included in the model, only sex remained significantly associated with adenine levels. Although regression diagnostics suggested some deviation from normality of residuals, the analysis was retained as an exploratory approach to assess potential independent associations and should therefore be interpreted with caution.

Among the variables included in the model, only sex was independently associated with adenine concentrations. Male sex was associated with an estimated increase of 30.02 µmol/L in adenine concentration compared with female sex (β = 30.02; 95% CI: 9.33–50.70; *p* = 0.0047). No significant associations were observed for age, LVEF category, or anemia status. These findings suggest that sex-related biological differences may contribute to variation in adenine metabolism among patients with CHF.

## 4. Discussion

In this cross-sectional cohort of patients with CHF, anemia was present in approximately 40% of participants, and disturbances in iron handling were evident even beyond hemoglobin-defined anemia. Serum iron was significantly lower in CHF than in controls, while ferritin did not differ significantly, which is consistent with the concept of functional iron deficiency in CHF, reflecting reduced iron bioavailability despite preserved or increased iron stores. This pattern is commonly linked to inflammation and hepcidin-mediated restriction of iron absorption and mobilization [18]. Clinically, this is relevant, as iron deficiency in heart failure is considered a therapeutic target independent of hemoglobin, and contemporary guidelines recommend intravenous iron therapy in symptomatic patients with iron deficiency [19,20]. Evidence from randomized trials and meta-analyses indicates that intravenous iron, particularly ferric carboxymaltose and ferric derisomaltose, improves symptoms and reduces heart failure-related hospitalizations, although its effect on mortality remains less consistent [21,22,23].

In addition to disturbances in iron metabolism, alterations in purine metabolism were observed in patients with CHF. In the present study, adenine levels were higher in CHF patients compared with controls, whereas other purine metabolites did not differ significantly between groups. However, given the number of comparisons performed, this finding should be interpreted cautiously. Previous studies have demonstrated that purine metabolism is closely linked to cellular energy metabolism and may be altered under conditions of tissue hypoxia and impaired energetics, which are characteristic features of chronic heart failure [24,25,26,27]. Under such conditions, accelerated degradation of adenosine triphosphate can lead to the accumulation of purine intermediates, reflecting increased nucleotide turnover and metabolic stress.

Correlation analyses did not demonstrate statistically significant associations between hemoglobin levels and purine metabolites in the overall CHF cohort or in subgroup analyses. While this may suggest a lack of a strong relationship between purine metabolism and anemia severity, these findings should be interpreted with caution, as the study may have been underpowered to detect more subtle associations. Similar considerations apply to subgroup analyses, where the number of comparisons increases the potential for both type I and type II errors. Importantly, the primary hypothesis of the present study was not supported by the data. Although we initially hypothesized that disturbances in purine metabolism might contribute to anemia development in CHF through interactions with iron metabolism and erythropoiesis, no significant associations were identified between hemoglobin levels and any of the investigated purine metabolites. Furthermore, adenine, the only metabolite that differed significantly between CHF patients and controls, was not independently associated with anemia status in the multivariable analysis. These findings suggest that alterations in purine metabolism may not represent a major determinant of anemia in CHF and instead may reflect broader metabolic disturbances associated with chronic heart failure itself.

Multivariable analysis further indicated that adenine levels were not significantly associated with anemia status, age, or LVEF category, whereas sex emerged as the only independent predictor of adenine concentration. Male sex was associated with significantly higher adenine levels compared with female sex, suggesting that biological sex may contribute to variability in purine metabolism among patients with CHF. Sex-related differences in hormonal regulation, oxidative stress responses, body composition, and purine metabolic pathways have been reported previously and may partially explain this observation. Although the present study was not specifically designed to investigate sex-dependent metabolic profiles, this finding highlights the potential importance of sex as a determinant of adenine metabolism and warrants further investigation in future studies. However, the overall explanatory power of the model was low (R^2^ = 0.049), indicating that these clinical variables account for only a small proportion of variability in adenine levels. In addition, deviation from normality of residuals suggests that the results of the regression analysis should be interpreted with caution. Taken together, these findings suggest that alterations in purine metabolism in CHF may reflect broader systemic metabolic processes rather than mechanisms directly related to anemia, although this hypothesis requires further investigation.

Additional correlation analyses suggested that purine metabolites were associated with ferritin levels in patients with CHF and anemia, while associations between purine metabolites and oxidative stress markers were observed in patients without anemia. These patterns should be interpreted cautiously but may indicate that purine metabolism reflects metabolic and oxidative stress pathways rather than processes specifically linked to anemia severity.

Several limitations should be considered when interpreting the results of this study. First, the cross-sectional design does not allow causal relationships between iron metabolism, purine metabolism, and anemia to be established. Second, the study was conducted at a single center with a relatively limited sample size, including a relatively small control group, which may reduce statistical power and limit the generalizability of the findings. Third, multiple statistical comparisons were performed, increasing the risk of type I error despite the use of correction methods. Fourth, potential confounding factors, including renal function, medication use, and comorbid conditions, may influence purine metabolism and oxidative stress markers.

Despite these limitations, the present study provides additional insight into metabolic alterations in patients with CHF. The findings suggest that disturbances in purine metabolism may occur alongside abnormalities in iron handling and oxidative stress. While no clear association with anemia severity was identified, the observed patterns are biologically plausible and consistent with existing evidence on metabolic dysregulation in chronic heart failure. Further studies with larger sample sizes and longitudinal designs are needed to clarify the role of purine metabolism in the pathophysiology of CHF and its potential clinical relevance.

The finding of lower LPO levels in CHF patients compared with controls, despite higher AOPP concentrations, appears counterintuitive and requires cautious interpretation. CHF is generally considered a condition associated with increased oxidative stress; however, different oxidative stress markers may reflect distinct biochemical processes and may not change in parallel. AOPPs primarily reflect protein oxidation and may be more sensitive to chronic inflammatory and oxidative conditions, whereas spectrophotometric LPO measurements may be influenced by assay specificity, sample handling, antioxidant status, and pharmacological treatment. In particular, medications commonly used in CHF, including statins, ACE inhibitors, beta-blockers, SGLT2 inhibitors, ARNIs, and diuretics, may modify oxidative stress pathways and could potentially affect LPO measurements. Therefore, the lower LPO values observed in CHF patients should not be interpreted as evidence of reduced oxidative stress, but rather as a marker-specific finding that requires confirmation using more specific lipid peroxidation assays.

### Limitations

An additional limitation of this study is the relatively broad age distribution of the study population. Age-related physiological changes and the increased prevalence of comorbid conditions in older individuals may influence purine metabolism, iron homeostasis, oxidative stress markers, and hematological parameters. Although age was included as a covariate in the multivariable regression analysis, residual confounding related to age and age-associated multimorbidity cannot be completely excluded. Several potentially important confounding factors, including chronic kidney disease, diabetes mellitus, inflammatory status, medication use, and overall comorbidity burden, were not systematically assessed and therefore could not be included in the multivariable analysis. As a result, residual confounding cannot be excluded, and the observed associations should be interpreted with caution. Future studies incorporating more comprehensive clinical characterization of participants are warranted. A major limitation of the present study is the absence of detailed data on pharmacological treatment. Medications commonly used in CHF and related comorbidities, including diuretics, statins, ACE inhibitors, beta-blockers, SGLT2 inhibitors, ARNIs, allopurinol, febuxostat, and iron supplementation, may influence purine metabolism, uric acid levels, iron indices, and oxidative stress markers. Because these data were not systematically available for all participants, medication use could not be tabulated or included in the multivariable models. Therefore, treatment-related residual confounding cannot be excluded.

## Figures and Tables

**Table 1:** Comparison of hematological, iron metabolism, purine metabolites, and oxidative stress parameters between control subjects and patients with CHF.

Parameter	Control (*n* = 29), Median (IQR)	CHF (*n* = 176), Median (IQR)	*p*-Value
Serum iron (µmol/L)	16.2 (13.5–19.8)	11.5 (6.9–17.5)	0.001
Ferritin (µg/L)	68.0 (49.0–115.9)	100.1 (39.4–168.1)	0.123
Hemoglobin (g/L)	132.0 (128.0–141.0)	132.0 (111.5–147.0)	0.579
Platelets (×10^9^/L)	288.0 (234.8–315.0)	225 (186.0–269.5)	0.001
Guanine (µmol/L)	186.0 (161.0–208.0)	195.0 (155.0–261.0)	0.242
Hypoxanthine (µmol/L)	142.0 (116.0–168.0)	164.0 (123.5–214.0)	0.058
Adenine (µmol/L)	105.0 (82.0–131.0)	135.0 (92.5–171.0)	0.009
Xanthine (µmol/L)	171.0 (152.0–192.0)	184.0 (140.5–238.5)	0.108
Uric acid (µmol/L)	194.0 (172.0–228.0)	213.0 (160.0–273.0)	0.232
Lipid peroxidation products (LPO)	0.215 (0.175–0.242)	0.157 (0.119–0.258)	0.021
Advanced oxidation protein products (AOPPs)	0.133 (0.086–0.200)	0.194 (0.135–0.277)	0.008

**Table 2:** Comparison of hematological, iron metabolism, purine metabolites, and oxidative stress parameters across CHF phenotypes.

Parameter	HFpEF (*n* = 51)	HFmrEF (*n* = 64)	HFrEF (*n* = 61)
Serum iron (µmol/L)	11.4 (6.0–17.2) ^ab^	13.9 (8.2–19.6) ^a^	10.0 (6.5–14.9) ^b^
Ferritin (µg/L)	60.0 (15.2–154.1) ^a^	108.8 (49.4–156.3) ^ab^	119.0 (51.9–187.6) ^b^
Hemoglobin (g/L)	116.0 (102.0–135.5) ^a^	137.0 (115.8–151.3) ^b^	138.0 (120.0–147.3) ^b^
Platelets (×10^9^/L)	261.0 (208.5–317.0) ^a^	222.0 (187.8–260.3) ^ab^	205.0 (177.8–243.0) ^b^
Guanine (µmol/L)	191.0 (161.5–255.5)	195.0 (151.8–251.5)	199.0 (158.0–273.3)
Hypoxanthine (µmol/L)	164.0 (122.0–206.5)	157.5 (124.0–201.8)	168.0 (129.0–228.0)
Adenine (µmol/L)	138.0 (86.0–171.0)	128.5 (98.8–163.5)	137.0 (94.0–191.5)
Xanthine (µmol/L)	191.0 (154.0–225.5)	175.5 (131.8–236.0)	197.0 (149.0–254.3)
Uric acid (µmol/L)	218.0 (163.5–261.0)	207.0 (150.8–246.0)	213.0 (166.0–291.8)
Lipid peroxidation products (LPO)	0.160 (0.119–0.265)	0.160 (0.114–0.270)	0.170 (0.130–0.259)
Advanced oxidation protein products (AOPPs)	0.210 (0.149–0.390)	0.200 (0.131–0.271)	0.180 (0.134–0.235)

Note: Values sharing the same superscript letter are not significantly different (Dunn’s multiple comparisons test, *p* < 0.05).

**Table 3:** Comparison of hematological, iron metabolism, purine metabolites, and oxidative stress parameters in CHF patients with and without anemia and control group.

Parameter	CHF with Anemia (*n* = 71)	CHF Without Anemia (*n* = 105)	Control (*n* = 29), Median (IQR)
Serum iron (µmol/L)	7.1 (4.6–12.4) ^a^	13.8 (9.9–19.6) ^b^	16.2 (13.5–19.8) ^b^
Ferritin (µg/L)	63.6 (19.3–185.0)	116.1 (59.2–162.6)	68.0 (49.0–115.9)
Hemoglobin (g/L)	106.0 (96.0–115.0) ^a^	144.0 (136.0–153.0) ^b^	132.0 (128.0–141.0) ^c^
Platelets (×10^9^/L)	243.0 (189.0–290.0) ^ab^	212.0 (186.0–261.0) ^b^	288.0 (234.8–315.0) ^a^
Guanine (µmol/L)	184.0 (158.0–255.5)	201.0 (156.0–262.0)	186.0 (161.0–208.0)
Hypoxanthine (µmol/L)	158.0 (125.0–202.5)	167.0 (124.0–217.0)	142.0 (116.0–168.0)
Adenine (µmol/L)	134.0 (93.0–164.0) ^ab^	135.0 (92.0–179.0) ^b^	105.0 (82.0–131.0) ^a^
Xanthine (µmol/L)	182.0 (137.5–229.0)	191.0 (149.0–242.0)	171.0 (152.0–192.0)
Uric acid (µmol/L)	209.0 (162.0–267.5)	213.0 (156.0–273.0)	194.0 (172.0–228.0)
Lipid peroxidation products (LPO)	0.160 (0.137–0.266)	0.160 (0.112–0.256)	0.215 (0.175–0.242)
Advanced oxidation protein products (AOPPs)	0.180 (0.137–0.290) ^ab^	0.210 (0.135–0.272) ^a^	0.133 (0.086–0.200) ^b^

Note: Data are presented as median (IQR). Pairwise comparisons were performed using Dunn’s post hoc test with Holm adjustment following a significant Kruskal–Wallis test. Groups sharing at least one common superscript letter are not significantly different from each other, whereas groups with no common superscript letters differ significantly (*p* < 0.05).

**Table 4:** Multivariable linear regression model evaluating independent predictors of adenine concentration in patients with chronic heart failure.

Variable	β (Estimate)	95% CI	*p*-Value
Age	0.38	−0.57 to 1.33	0.435
Sex (male vs. female)	30.02	9.33 to 50.70	0.0047
LVEF category	2.53	−10.10 to 15.16	0.693
Anemia (yes vs. no)	3.28	−17.61 to 24.17	0.757

Note: Model statistics: R^2^ = 0.049. Dependent variable: adenine concentration. β coefficients represent the estimated change in adenine concentration associated with each predictor while holding the remaining variables constant. For categorical variables, β represents the difference relative to the reference category.

## Data Availability

The datasets generated and analyzed during the current study are not publicly available due to patient confidentiality and institutional regulations but are available from the corresponding author upon reasonable request.

## Supplementary Materials

The following supporting information can be downloaded at: https://www.mdpi.com/article/10.3390/metabo16060432/s1, Table S1: Laboratory parameters in patients with chronic heart failure stratified by phenotype and anemia status.

## Abbreviations

The following abbreviations are used in this manuscript:

CHF	Chronic heart failure
HF	Heart failure
HFpEF	Heart failure with preserved ejection fraction
HFmrEF	Heart failure with mildly reduced ejection fraction
HFrEF	Heart failure with reduced ejection fraction
ATP	Adenosine triphosphate
LPO	Lipid peroxidation products
AOPP	Advanced oxidation protein products
IV	Intravenous
IQR	Interquartile range
