# Supplementary material for: Purine Metabolism Alterations in Patients with Chronic Heart Failure: A Cross-Sectional Study of Associations with Iron Status, Oxidative Stress, and Anemia
Source: Metabolites. 2026 Jun 22;16(6):432. doi: 10.3390/metabo16060432 (PMC13302895; doi:10.3390/metabo16060432)
Supplement: Supplementary file 1 [file metabolites-16-00432-s001.zip › metabolites-4321155-supplementary.pdf]

Supplementary Materials

Supplementrary Table S1. Laboratory parameters in patients with chronic heart failure stratified by phenotype and anemia status

Parameter	HFpEF with an (n=29)	HFpEF without an (n=22)	HFmrEF with an (n=22)	HFmrEF without an (n=42)	HFrEF with an (n=20)	HFrEF without an (n=41)
Serum iron (µmol/L)	6.6 (3.6–11.5)[a]	16.1 (11.6–21.9)[b]	7.8 (6.1–16.3)[ab]	15.7 (11.1–20.4)[b]	6.3 (4.6–10.2)[c]	12.0 (7.5–16.5)[b]
Ferritin (µg/L)	32.5 (12.1–143.8)[a]	98.8 (52.1–162.8)[ab]	75.4 (32.0–305.7)[ab]	113.2 (66.83–150.5)[ab]	78.9 (36.7–193.3)[ab]	131.0 (62.3–187.7)[b]
Hemoglobin (g/L)	104.0 (96.0–112.5)[a]	143.0 (131.8–152.3)[b]	111.5 (84.0–116.3)[a]	148.0 (137.5–153.5)[b]	108.0 (97.0–123.0)[a]	143.0 (137.5–154.0)[b]
Platelets ($\times 10^9$/L)	281.0 (230.5–379.0)[a]	213.0 (185.3–273.0)[ab]	235.0 (158.3–262.0)[ab]	219.0 (188.8–261.0)[b]	205.5 (175.5–247.5)[b]	205.0 (177.4–241.5)[b]
Guanine (µmol/L)	183.0 (146.0–246.0)	219.0 (172.0–291.5)	189.0 (156.5–277.5)	195.0 (125.0–248.0)	193.0 (145.3–310.5)	206.0 (155.0–265.5)
Hypoxanthine (µmol/L)	154.0 (115.0–197.0)	173.0 (126.8–244.8)	155.5 (126.0–216.0)	158.5 (117.3–201.8)	167.0 (127.3–259.3)	168.0 (126.5–222.5)
Adenine (µmol/L)	137.0 (78.5–162.0)	138.5 (90.0–193.0)	128.0 (99.5–160.8)	128.5 (90.5–171.3)	135.5 (93.0–194.0)	140.0 (104.5–191.0)
Xanthine (µmol/L)	182.0 (140.0–218.0)	195.0 (161.0–244.3)	179.5 (137.0–247.8)	174.0 (126.3–236.0)	186.5 (132.5–284.3)	202.0 (154.5–252.0)
Uric acid (µmol/L)	192.0 (149.5–251.5)	239.5 (182.3–298.3)	210.0 (172.5–310.3)	195.5 (139.3–246.0)	232.0 (169.0–294.8)	207.0 (159.5–292.5)
Lipid peroxidation products (LPO)	0.160 (0.135–0.275)	0.140 (0.090–0.238)	0.160 (0.138–0.268)	0.155 (0.110–0.275)	0.195 (0.133–0.268)	0.160 (0.120–0.250)
Advanced oxidation protein products (AOPP)	0.200 (0.150–0.390)	0.235 (0.140–0.423)	0.195 (0.130–0.295)	0.210 (0.128–0.275)	0.155 (0.113–0.193)	0.210 (0.145–0.255)

Note: Values sharing the same superscript letter are not significantly different (Dunn's multiple comparisons test, $p < 0.05$).
